# Functional and Molecular Characterization of Extracellular Vesicles Enriched in Exosomes Released by Bone Marrow Mesenchymal Stromal Cells Exposed to IFNγ in Combination with Autophagy Modulators Tamoxifen or Chloroquine

**DOI:** 10.3390/ncrna12010001

**Published:** 2025-12-24

**Authors:** Vladimir Beljanski, Maria J. Moreno Hollweg, Renee Potens, Tanner Blaylock, Andres B. Irausquin, Nikhila Paleati, Lubov Nathanson

**Affiliations:** 1 Dr Kiran C. Patel College of Allopathic Medicine, Nova Southeastern University, Davie, FL 33314, USA; tannerblaylock@usf.edu; 2 Cell Therapy Institute, Dr Kiran C. Patel College of Allopathic Medicine, Nova Southeastern University, Davie, FL 33314, USA; mmorenoh@nova.edu (M.J.M.H.); rp641@nova.edu (R.P.); ai240@nova.edu (A.B.I.); 3 Department of Biological Sciences, Halmos College of Arts and Sciences, Nova Southeastern University, Fort Lauderdale, Davie, FL 33314, USA; np1037@mynsu.nova.edu; 4 Institute for Neuroimmune Medicine, Dr Kiran C. Patel College of Osteopathic Medicine, Nova Southeastern University, Davie, FL 33314, USA; lnathanson@nova.edu

**Keywords:** mesenchymal stromal cells, extracellular vesicles, exosomes, immunoregulation, autophagy, tamoxifen, chloroquine, microRNAs, gene regulation

## Abstract

**Background/Objectives**: Bone marrow mesenchymal stromal cells (MSCs) are therapeutic cells that adopt an immunomodulatory phenotype when exposed to pro-inflammatory cytokines. Recent research efforts uncovered that many therapeutic benefits of MSCs can be attributed to the secretion of extracellular vesicles (EVs) such as exosomes, small membrane vesicles of endocytic origin present in the cellular secretome. EVs’ formation and release are impacted by the autophagy pathway, which recycles proteins and organelles via lysosomal degradation. **Methods**: To evaluate how modulation of autophagy affects properties of MSC EVs enriched in exosomes under pro-inflammatory conditions, we treated the cells with either tamoxifen (TX) or chloroquine (CQ), two drugs known to stimulate or inhibit autophagy, respectively, together with IFNγ. MSC EVs enriched in exosomes were then purified from serum-free media, and their immunoregulatory properties were evaluated ex vivo using activated CD4 T cells; small RNA sequencing was also conducted to determine EVs’ microRNA content. **Results**: Our data indicate that MSCs treated with CQ + IFNγ yield EVs that possess somewhat higher capacity to decrease T cell proliferation compared to other EVs. Small RNA sequencing revealed that, although similar microRNAs were found in EVs isolated from all treated cells, the treatments exerted more effect on the levels of multiple microRNAs that are known to regulate either cancer or inflammation-related biological pathways in target cells. **Conclusions**: Overall, we conclude that the co-treatment of MSCs with TX or CQ in the presence of pro-inflammatory cytokine IFNγ has the potential to modulate microRNA content of EVs, potentially affecting biological properties of such EVs and their effect on target cells.

## 1. Introduction

Bone marrow mesenchymal stem/stromal cells (MSCs) are progenitor cells that can be differentiated into cells of mesodermal origin, such as adipocytes, osteocytes, and chondrocytes [1]. Due to their abundance and straightforward expansion protocols, MSCs have been recognized as promising candidates for cellular therapy. The pleiotropic biological effects of MSCs allow for their use in multiple conditions, including those caused by excessive inflammation. MSCs possess high immunomodulatory capacity; when exposed to pro-inflammatory cytokines, MSCs typically adopt an anti-inflammatory phenotype by secretion of immunomodulatory cytokines (such as IL-6) and by increased surface expression of the PD-L1 molecule [2].

The original mechanism of MSC-based therapies was thought to be direct cell replacement [3]. However, labeling studies revealed that only a few MSCs engrafted at injury sites, and these studies additionally revealed that intravenously administered MSCs achieved caught in the capillaries of the lung, where they were subsequently cleared [4,5]. Despite such limitations, MSC treatments continue to yield therapeutic benefits in multiple disease models, leading the field of cell therapies to adopt a viewpoint in which MSCs facilitate tissue repair mostly via paracrine factor secretion, which stimulates survival and repair of host cells [6]. Therefore, it was suggested that the therapeutic benefits of MSCs originate from secreted factors rather than from cell differentiation and tissue integration. Subsequent studies revealed that the MSC secretome contributes to tissue regeneration by inducing a shift from a pro-inflammatory to an anti-inflammatory state at the site of injury [7,8]. These and other observations support the development of secretome-based cell-free therapies, which circumvent the risks associated with cell-based therapies, such as immune rejection and/or accumulation of mutations due to genetic instability [9,10].

Most nucleated cells possess intracellular vesicle sorting mechanisms that produce extracellular vesicles (EVs), microvesicles of >200 nm, and exosomes of 70–150 nm diameter [11]. Exosomes can transfer the contents of cells from which they originate to neighboring cells, influencing the signaling pathways of the latter. For example, exosomes can be utilized by immune cells for communication with other immune cells [12] or with tumor cells [13]. Evidence suggests that MSC exosomes alone are responsible to a significant extent for the therapeutic effects previously attributed to MSCs [14]. MSC exosomes contain both microRNAs (miRs) and proteins with anti-inflammatory and anti-apoptotic properties, which are then taken up by target cells, leading to modulation of target cell signaling [15].

Macroautophagy (referred to here on as “autophagy”) is an evolutionarily conserved lysosomal catabolic pathway whose major role is the maintenance of cellular homeostasis (reviewed in [16]). Autophagy is frequently upregulated in response to extracellular or intracellular stress, including starvation, growth factor deprivation, endoplasmic reticulum stress, and infection [17,18]. Key roles of autophagy in exosome secretion were uncovered in studies of molecular and functional crosstalk between exosome release and autophagy pathways. Pre-exosomal multivesicular bodies (MVBs) co-localize with autophagy protein LC3 upon treatment of cells with autophagy inducers such as rapamycin or starvation [19]. In addition, autophagy induction or overexpression of LC3 inhibited exosome release, suggesting that under conditions that stimulate autophagy, MVBs are directed to the autophagic pathway and degraded, resulting in inhibition of exosome release [20]. Several studies have also revealed that RNA processing machinery assembles next to MVBs and autophagy regulates miRs activity by promoting selective degradation of two key RNA silencing proteins, DICER and AGO2 [21].

We utilized a subtype of MSCs called MIAMI cells previously to observe changes in the mRNA and protein levels of several genes involved in MSC-mediated immunomodulation upon the addition of the autophagy stimulator tamoxifen (TX) or autophagy inhibitor chloroquine (CQ) [21]. Interestingly, we also discovered that the treatments influenced the cytosolic levels of various non-coding RNAs and a group of miRs targeting immunomodulatory genes [21,22]. To investigate the impact of the autophagy modulators CQ and TX on the miR content of MSC exosomes (within EVs), we exposed MSCs to these modulators in the presence or absence of IFNγ. As autophagy has the potential to regulate the content of exosomal RNAs, we also examined the miR content of exosomes isolated from the pre-treated MSCs. Additionally, we assessed the effects of IFNγ, CQ, and TX treatments on EVs’ functional properties by evaluating their effect on T cell proliferation. Our results indicate only a modest effect of the treatments on EVs ability to decrease activated T cell proliferation, and the majority of differentially regulated miRs belonged to either cancer or inflammation-related pathways.

## 2. Results

### 2.1. Assessment of Autophagy Markers in MSCs

**Figure 1:**
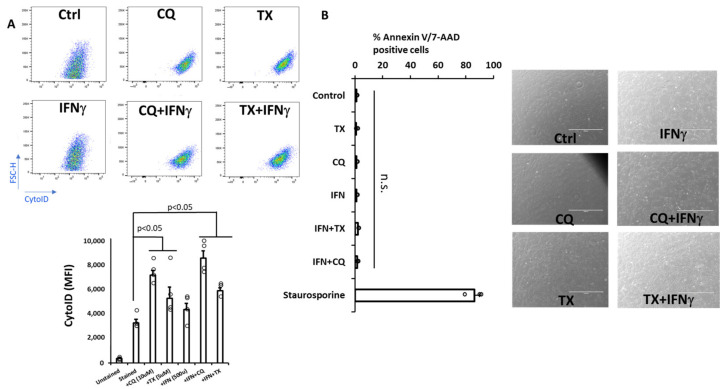
TX or CQ treatments cause accumulation of autophagic vehicles in MSCs without cytotoxicity or upregulation of apoptotic markers. MSCs were treated as indicated for 48 h to evaluate markers of apoptosis and autophagy (n = 3–4). (**A**) Assessment of autophagic vehicles using Cyto-ID staining of autophagic vesicles by flow cytometry and its quantification (**bottom**); (**B**) Annexin V and 7AAD staining of cells after exposure to TX, CQ, IFNg, or combinations (**left**) and light microscopy assessment of cells upon treatment (bar = 1000 mm); staurosporine was used as a positive control. Circles around bar graphs indicate individual data points (n = 3–5). Error bars represent the standard error between the replicates. n.s.: no statistical significance between the groups to the left of the line.

Similarly to our previous approach to stimulating and inhibiting autophagy in MIAMI cells, we exposed MSCs to either CQ or TX alone or in the presence of IFNg [22,23]. Accumulation of autophagic vesicles in cells due to the pathway stimulation with TX or inhibition of autophagosome degradation with CQ was confirmed via immunoblotting for increased LC3B/actin ratios (data not shown) and 2–3-fold increases in CytoID signal (Figure 1A). The absence of treatment-related toxicity (i.e., apoptosis) was confirmed via AnnexinV/7-AAD staining and by visual observation of adherent cells (Figure 1B).

### 2.2. Isolation and Characterization of MSCs’ EVs Enriched in Exosomes upon TX or CQ Treatments

It has been shown previously that exposure of MSCs to pro-inflammatory cytokines enhances the immunoregulatory potential of the cells [24,25]. Because MSCs’ immunoregulatory properties are mediated in part via secretion of EVs, and autophagy may contribute to biological functions of the secreted exosomes, we isolated EVs enriched in exosomes (referred to as exosomes from this point on) from MSCs exposed to IFNγ with or without CQ or TX. MSCs grown in serum-free media were exposed to CQ, TX, IFNg, CQ + IFNγ, or TX + IFNγ for 48 h; cell media was collected twice, at 24 and 48 h, and pooled together. Cell media fractions containing treatment-modified exosomes were pooled together, centrifuged to remove cell debris, concentrated, and exosome preps were obtained by separating concentrated media using size exclusion chromatography on a Sephacryl 400 column as described in the Methods section. We did not use ultracentrifugation to avoid exposing EVs to high g-forces and to minimize rupture. Subsequently, collected exosomes were resuspended in PBS and characterized using both biochemical and functional assays (see below).

**Figure 2:**
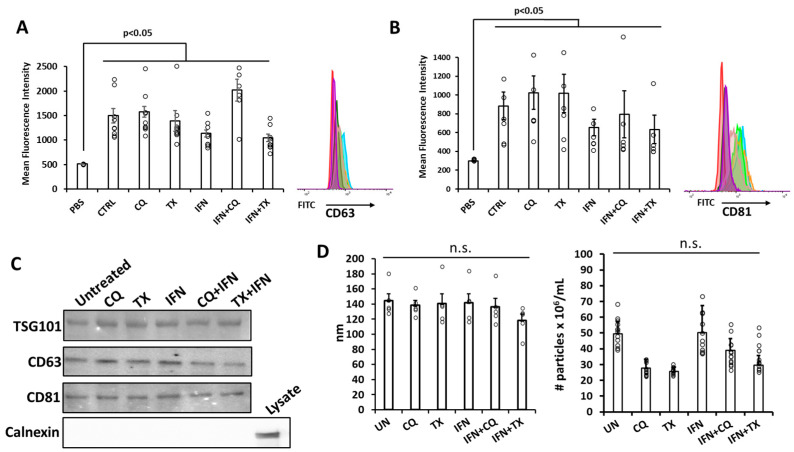
Characterization of EVs enriched in exosomes isolated from MSCs treated with TX, CQ, IFNg, or combinations. Flow cytometry assessment of exosomal CD63 (**A**) and CD81 (**B**) markers. Exosomes isolated and purified as described in Materials and Methods were combined with either CD63 or CD81 magnetic beads overnight at 4 °C, after which CD63 or CD81 antibody was added. This mixture was analyzed on a flow cytometer using 488/519 nm excitation/emission wavelengths. Representative overlayed flow histograms are shown next to the graphs; (**C**) immunoblotting for expression of exosomal markers; (**D**) Circles around bar graphs indicate individual data points (n = 5–8). NanoSight analysis of the average exosome sizes. Error bars represent the standard error between the replicates. n.s.: no statistical significance between the groups below the line.

FPLC fractions enriched in exosomes were [24,25] first characterized using positive selection capture microbeads recognizing the tetraspanin CD63 and CD81 proteins on intact exosomes. Upon sample equalization based on total protein and incubation with the magnetic microbeads containing either CD63 or CD81 antibodies, the magnetically labeled exosomes were retained using a magnet, incubated with appropriate fluorescent antibodies, and finally, antibody-stained exosomes were analyzed using flow cytometry; fluorescence intensity data were compared to the vehicle (PBS). We found that exposure of MSCs to treatments did affect the expression of the two markers, although such differences do not appear to be statistically significant (Figure 2A,B). Immunoblotting for exosomal markers confirmed expected signals for CD63, CD81, and TSG101 and the absence of signal for calnexin, indicating lack of contamination of exosomes with ER membranes (Figure 2C and Figure S2).

Nanoparticle tracking analysis using NanoSight technology (Malvern Panalytical, Westborough, MA, USA) combines the properties of both laser light scattering microscopy and Brownian motion to obtain size distributions of particles in liquid suspension, such as PBS [26]. To determine whether the treatments affect the size distribution of exosomes, we measured their sizes and concentration using Nanosight N300, which showed that purified exosomes had an average size range of between 50 and 180 nm and a concentration of 0.3 to 1.8 × 10^10^ particle/mL with no statistically significant differences between the different treatments (Figure 2D).

**Figure 3:**
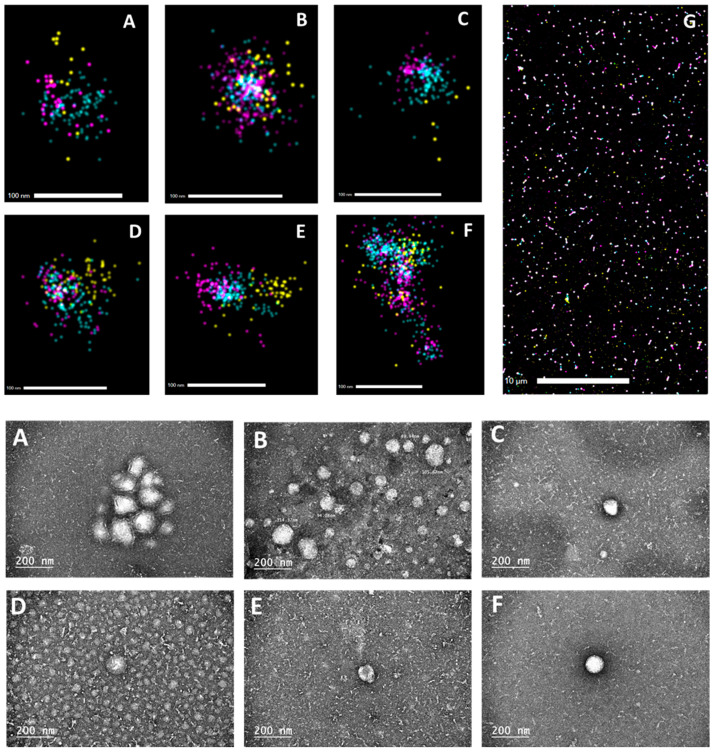
(**Top**): Three-color dSTORM visualization of exosomal tetraspanins on the surface of individual particles. dSTORM imaging was performed on EVs enriched in exosomes using emissions from CD9-ATTO488 (green), anti-CD63-Cy3 (orange), and anti-CD81-AlexaFluor647 (red). Representative images are shown on a scale of 10 mM or 100 nm as indicated on panels. A representative wide-field image of exosomes stained with tetraspanin-targeting antibodies isolated from vehicle-treated MSCs is shown in (**G**). For all image acquisitions, three independent exposures were taken with >500 individual EVs identified via antibody staining. (**Bottom**): transmission electron microscopy of EVs isolated from supernatants of MSCs. EVs enriched in exosomes were isolated and purified using centrifugation, ultrafiltration, and size exclusion chromatography, and were applied onto copper grids. Subsequently, they were stained with uranyl acetate and examined. EVs were isolated from the serum-free media of MSC treated with the following: (**A**) vehicle, (**B**) TX, (**C**) CQ, (**D**) IFNγ, (**E**) TX + IFNγ, (**F**) CQ + IFNγ.

To provide further evidence for tetraspanins on exosomes, three different tetraspanins on the surface of individual exosomes were imaged using dSTORM. Total exosomes isolated from three independently isolated fractions for each MSC treatment were pooled together and seeded onto glass slides and subjected to dSTORM imaging using parameters described in the Methods section. At low resolution, clustering of the three markers, CD63, CD81, and CD9, within ≤10 mm space was apparent and used to select individual exosomes (Figure 3A–G, top panels). The distribution of tetraspanins in imaged exosomes was similar between the treatments (Figure S1).

The shape and structure of isolated exosomes were also assessed by TEM, confirming “cup-shaped” vesicles as the dehydrated, negatively stained samples used for TEM shrink during preparation, causing a “cup-shaped” morphology (Figure 3A–F, bottom panels).

### 2.3. The Effects of TX or CQ on MSC Exosomes’ Capacity to Decrease T Cell Activation and Proliferation

**Figure 4:**
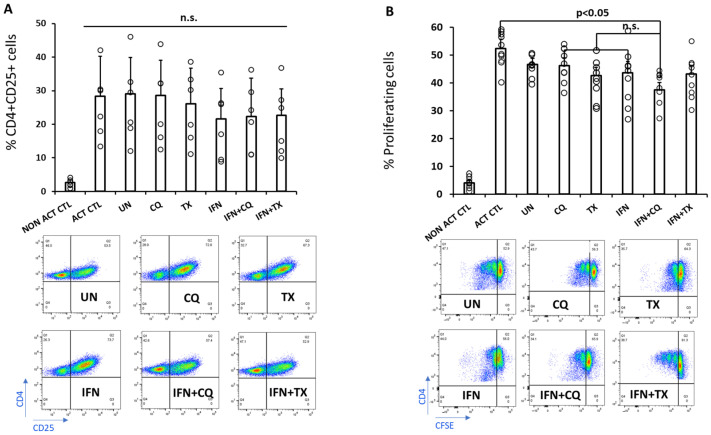
The effects of extracellular vesicles isolated from supernatants of MSCs treated (or not) with TX, CQ, IFNγ, or combinations (as indicated on panels) on the expression of CD25, CD4 T cell activation marker (**A**), and reduction in CD4 T cell proliferation ex vivo (**B**). 50 mg of exosomes were co-cultured with modified exosomes as indicated below the graphs for 3 (**A**) or 4 days (**B**) to evaluate their effect on the cell proliferation and expression of the activation marker CD25. Flow cytometry analysis was used to quantify the events, and examples of flow diagrams are shown at the bottom. Graphs are a representation of n = 8 (proliferation) and n = 5 (CD25 marker expression) independent experiments. Circles around bar graphs indicate individual data points. Error bars represent the standard error between the replicates. N.s.: no statistical significance between groups below the line.

To determine whether manipulation of autophagy via CQ or TX treatments, in addition to IFNg, impacts on exosomal immunomodulatory capacity, treatment-modified exosomes were incubated with CD4 T cells isolated from healthy donors activated with anti-CD3/CD28 antibodies. CD4 T cell activation was measured by assessing CD25 surface expression and CD4 T cell proliferation using flow cytometry. Activated CD4 T cells co-cultured with modified MSC exosomes for 3 days displayed no changes in CD25 expression (Figure 4A). We next evaluated the effects of treatment-modified exosomes on CD4 T cell proliferation by staining of CD4 T cells with CFSE followed by flow cytometry-based analysis on day 4. Incubation of T cells with exosomes decreased T cell proliferation by 5–10% compared to vehicle control (Figure 4B). This decrease was comparable to what was observed using exosomes isolated from MSCs treated with CQ or IFNγ. MSC treatment with TX or TX + IFNγ slightly decreased CD4 T cell proliferation, but the highest capacity to decrease T cell proliferation was observed for the exosomes isolated from MSCs treated with CQ + IFNγ (~15% compared to untreated exosomes). Such data indicate that exposure of MSCs to IFNγ in the presence of CQ contributes to the secretion of exosomes with moderately increased capacity to suppress CD4 T cell proliferation ex vivo.

### 2.4. Differential Expressions of Exosomal miRs Isolated from CQ, TX, IFNg, or Combination-Treated MSCs

**Figure 5:**
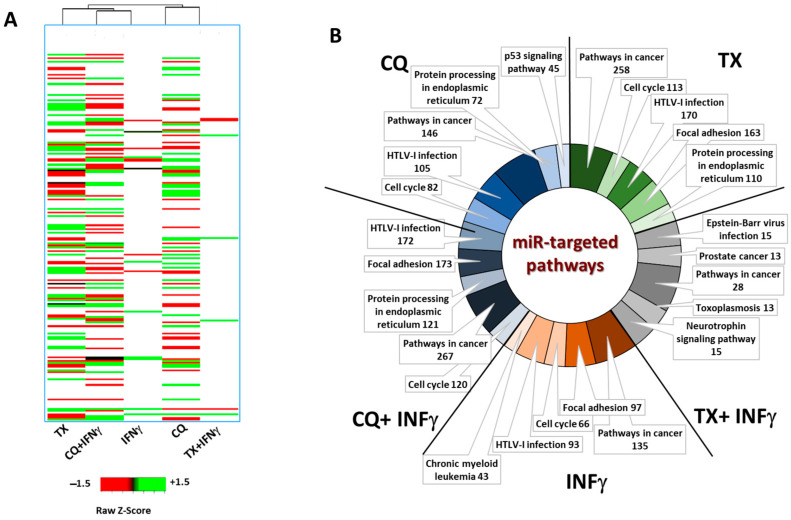
Analysis of differentially expressed exosomal miRs and their target pathways. (**A**) The heatmap shows unsupervised clustering of statistically significantly differentially regulated exosomal miRs resulting from different treatments. The miRs shown in the heatmap are derived from MSC exosomes (n = 3) treated as indicated on the bottom and were selected based on the following criteria: absolute fold change > 1.5 and *p* < 0.05. Missing values that did not pass these criteria are shown in white. (**B**) For each treatment, the differentially regulated miR-targeted pathways are indicated. Target genes that belong to multiple KEGG pathways were placed into each assigned pathway, and the number of target genes is written next to the pathway.

To determine how combining CQ or TX with IFNγ affects exosomal miRs expression, we isolated exosomes from MSCs exposed to IFNγ with or without CQ or TX. Subsequently, miRs were extracted from exosomes, and small RNA sequencing was performed. Differentially expressed miRs (DEmiRs) for each experimental group were normalized to those isolated from untreated MSCs, and fold change was calculated. Randomly selected 12 DEmiRs were utilized to validate RNA sequencing data (Figure S3). A total of 83 DEmiRs were identified in exosomes secreted by CQ-treated MSCs (44 upregulated and 39 downregulated) compared to untreated MSCs. Moreover, 140 DEmiRs (64 upregulated and 76 downregulated) were identified in the exosomes isolated from TX-treated MSCs, 19 DEmiRs (8 upregulated and 11 downregulated) were identified in INFγ -treated MSCs, 110 DEmiRs (58 upregulated and 52 downregulated) were identified in CQ + INFγ-treated MSCs, and 12 DEMs (8 upregulated and 4 downregulated) were identified in TX + INFγ-treated MSCs (Figure 5A). To evaluate potential TX, CQ, IFNγ, or combination-related changes in miR target genes and associated pathways (in target cells), the DEmiRs were imported into the miRNet database, which utilizes an experimentally validated miR-target interactions database. This allowed for the determination of genes that are targets of DEmiRs from our datasets, as well as pathways associated with such genes (Figure 5B). The pathways are based on the KEGG pathway database in the ‘Function Explorer’ module of the miRNet database. The only target pathway from the KEGG database that was common for each treatment group was Pathways in Cancer. HTLV-I infection and Cell signaling pathways were observed as potential targets in all treatment groups but TX + IFNγ. Potential differential targeting of Protein processing in the endoplasmic reticulum pathway was observed in CQ, TX, and CQ + IFNg treatment groups, whereas the focal adhesion pathway was observed in TX, IFNg, and CQ + IFNγ groups. The p53 signaling pathway was only observed as a potential target in the CQ group, and Epstein–Barr virus infection, Prostate cancer, Toxoplasmosis, and Neurotrophin signaling pathways were only observed in the TX group. This analysis, while only hypothetical, indicates that it is possible to fine-tune the effects of exosomes on pathways associated with cancer and infection-related diseases by using small molecules that modify cell stress-response mechanisms.

### 2.5. DEmiR Gene Targets and Their Biological Functions

Because miRs typically target multiple different mRNAs, we next examined the putative gene targets of exosomal miRs using experimentally validated gene target function in miRNet to create interconnections of DEmiRs and their targets. To further confirm cancer and/or inflammation-related roles of target genes, we visualized miRNA-Target Gene Pathway network utilizing miRNet’s ‘Network Viewer’ function using DEmiRs from each experimental group. To facilitate visualization of the DEmiR targets, the interacting nodes for CQ, TX, and CQ + INFγ groups (larger data sets) were pruned using parameters: degree > 3, betweenness > 100. Utilizing the function explorer application, all genes from the top 5 KEGG Pathways were queried using the hypergeometric test algorithm on the KEGG pathway database. Significant KEGG pathways relating to immune/inflammatory pathways were then chosen using the *p*-value cut-off <0.05 and highlighted as red nodes (Figure S4). The same procedure was followed to highlight significant KEGG pathways (*p*-value < 0.05) relating to cancer and highlighted as blue nodes. The size of the nodes is determined by the number of DEmiRs targeting genes, and the size of green squares (DEmiRs) is similarly based on the number of target genes. For this analysis, KEGG pathways such as Pathways in cancer, Cell cycle, MAPK signaling pathway, Wnt signaling pathway, and various cancer-related signaling pathways were considered as cancer-related pathways (based on their roles in either cell proliferation or apoptosis). Similarly, pathways such as HTLV-1 infection, Toxoplasmosis, Influenza A, and similar pathways were considered inflammation-related pathways. This allowed for the visualization of the target genes with respect to their roles in cell physiology. This data analysis indicates that most of the putative target genes of DEmiRs belong to either cancer or inflammation-related pathways; relative complexity for putative interactions of such targets is depicted in Figure S4. As noted above, this analysis is strictly hypothetical, and further experimental validation is needed for confirmation.

## 3. Discussion

While cell-based therapies have shown significant promise in treating various diseases, often outperforming conventional approaches, these therapies also come with limitations that hinder their clinical translation. In contrast, cell-free therapies based on EVs offer several compelling advantages, including low immunogenicity, minimal risk of infusion-related toxicity, ease of accessibility and storage, and an absence of tumorigenic and ethical concerns [27]. Importantly, EVs can recapitulate many of the therapeutic benefits of their parent cells by delivering biomolecules involved in tissue regeneration. However, significant gaps remain, especially concerning the systematic investigation of the relationship between modifications and downstream clinical applications.

Recently, EVs isolated from MSCs have gained significant attention for their potential in tissue repair therapies, offering a viable approach to cell-free treatment. However, the transition from promising preclinical studies to clinical applications has been hampered by significant limitations, including efficacy and production costs. Preconditioning, an adjunctive method in cell therapy, has shown promise to overcome such limitations by enhancing MSC proliferation, migration, and targeted differentiation [28]. Preconditioning can also stimulate exosome secretion and functionality by altering the molecular content of EVs [29]. TNFα and IFNγ are the most common cytokines used for MSC preconditioning, resulting in upregulation of key immunomodulatory molecules such as IDO, (PG)E-2, TGFβ, IL-10, and PD-L1, while the combination of both cytokines was shown to further increase the expression of immunomodulatory molecules such as IDO, PTGS-2, and iNOS [30]. Additionally, preconditioning can also include hypoxia, genetic modifications, pre-treatment with lipopolysaccharide, cytokines, or small molecules such as hydrogen peroxide or hydrogen sulfide (reviewed in [31]).

We tested here a novel approach to modulating the biological effects of MSC exosomes through the combined use of pro-inflammatory priming (i.e., exposure to IFNγ) and pharmacological modulators of autophagy. While previous studies have investigated the effects of small molecules on exosome secretion, much of this work has focused on quantitative outcomes, such as the number of extracellular vesicles released [32,33], without exploring the qualitative and functional consequences of these changes. In contrast, we sought to provide parallel molecular and functional characterization, allowing for the comparison of exosomal composition and biological activity. Our findings confirm that exosomes are heterogeneous entities, both in terms of cargo and surface marker expression [29,34,35]. The variability we observed in canonical exosomal markers such as CD63 and CD81 across treatment conditions mirrors recent proteomic analyses, indicating that marker expression is not uniform across all exosomes and may shift depending on cell state or treatment [36]. This reinforces current concerns in the field about the limitations of marker-based purification protocols, which may inadvertently exclude therapeutically relevant subpopulations of exosomes [37].

Functional assessment of these modified exosomes was performed to assess their ability to suppress CD4 T cell proliferation and expression of an activation marker, CD25. In T cell suppression assays, exosomes from IFNγ + CQ-treated MSCs demonstrated moderately enhanced suppression of CD4+ T cell proliferation, while others did not reach statistical significance. Similarly, no statistically significant data were observed for the expression of the CD4 T cell activation marker, CD25 [38].

Small RNA sequencing analysis revealed extensive treatment-induced changes in exosomal miRNA cargo. Both TX and CQ treatments resulted in distinct miRNA signatures, with TX predominantly affecting miRNAs targeting cell proliferation pathways, while CQ treatment modified miRNAs involved in both proliferation and inflammatory response regulation. Intriguingly, we observed substantial overlap between TX and CQ-induced changes in miRNA levels, suggesting common downstream effects of autophagy modulation. It was demonstrated previously that miRNAs carried within extracellular vesicles can regulate gene expression in distant tissues [39]. Our finding that the levels of numerous miRNAs can be modulated through combinatory treatments highlights the vast potential of the drugs in reshaping cellular communication, which is something that has not been extensively researched thus far. Interestingly, only about 10% of DEmiRs in our dataset were shown previously to be altered in exosomes due to cellular preconditioning. For example, miR-221 was increased in atorvastatin-treated MSC was shown to contribute to angiogenesis [40]; miR-140 was enriched in exosomes isolated from MSCs overexpressing oxygenase-1 and was shown to accelerate growth of osteoblasts [41]; miR-212 was overexpressed in MSCs and their exosomes and was shown to target release of pro-inflammatory mediators [42]; miR-210 was upregulated in MSCs preconditioned with hypoxia and was shown to enhance pro-angiogenic properties of MSC exosomes [43]; Both let-7b and miR-24 were found enriched in exosomes that underwent surface modification which promoted immunomodulation that was associated with increased macrophage M2 polarization [44]; adipose-derived MSCs overexpressing secreted exosomes enriched in miR-146a possessed enhanced suppression of acute myocarditis-induced apoptosis, inflammatory response, and fibrosis in a rat model of myocardial infraction [45]; miR-199a was found to target mTOR pathway when delivered via MSC exosomes leading to sensitization of hepatocellular carcinoma cells to cancer drugs [46]. Several miRs from our dataset, mir-145, mir-193b, mir-24, and mir-222 are known to modulate T cell function by regulating differentiation of T helper cell subsets, controlling T cell activation and apoptosis, and fine-tuning immune responses [47]. For example, miR-193b targets the MYB oncogene, but its role in other T cell functions can be context-dependent. In other immune contexts, miR-193b has been shown to be induced in macrophages by tumor cells and can promote tumor invasion, while exosomal miR-193b from dendritic cells can suppress liver transplant rejection by promoting regulatory T cells [48].

A critical finding emerged from the correlation analysis between miRNA composition and functional outcomes: changes in exosomal miRNA profiles did not directly predict T cell suppressive capacity. This observation carries significant implications for exosome engineering strategies, suggesting that miRNA cargo modification alone may be insufficient for enhancing therapeutic efficacy. Further, it indicates that other exosomal components, particularly exosome-delivered proteins, may play substantial roles in mediating immunomodulatory effects. While we did not delve into the effects of our treatments on exosomal proteins, future work needs to address potential changes in protein composition in modified exosomes. A limitation of the present study is that exosomes were derived from a single mesenchymal stem cell (MSC) line, which limits the generalizability of our findings. Therefore, the observed changes in exosomal miRNAs and their associated functional effects should be interpreted within the context of this limitation.

Finally, we did not observe opposing effects between TX and CQ, suggesting that autophagy alone may not fully account for the observed phenomena. Several factors may contribute to this outcome. These pleiotropic effects could converge on pathways that influence exosome biogenesis and cargo selection independently of autophagic flux. Second, it is increasingly recognized that autophagy modulators can have context-dependent effects depending on cell type, treatment duration, and metabolic state. In our experimental conditions, TX could be activating non-canonical or incomplete autophagy, whereas CQ could have affected exosome release due to impaired cargo clearance. This complexity between autophagic modulation and exosome output has been reported in prior studies [49,50].

Taken together, our results also underscore the limitations of using miRNA profiles as predictive biomarkers of exosome efficacy and point to the need for more integrative strategies that consider the full spectrum of exosomal cargo to understand the full potential of the latter.

## 4. Materials and Methods

### 4.1. Materials

TX and IFNγ were purchased from Sigma-Aldrich, and CQ was purchased from VWR. Cells, cell media, materials, and antibodies were purchased from various sources (as indicated below). Buffy coats were purchased from Continental Service Group Blood Bank (Fort Lauderdale) with K_2_EDTA as an anti-coagulant. Donor inclusion criteria were set at age 20–40 years with a BMI of 29 or less, non-smokers, and free of HIV, hepatitis B, hepatitis C, syphilis, West Nile virus, and ZIKA virus. To protect confidentiality, all specimens were assigned a unique identifier, a lot number, that is linked to the individual donor’s identifying information (such as name, contact information, etc.) The ID number linking each blood sample to an individual is kept electronically, password-protected, and only accessible by approved personnel at Continental. Samples sent to researchers were labeled with only the coded lot number and no personal identifying information.

### 4.2. T Cell Assays

PBMCs were isolated on the same day as collection. The buffy coat was diluted 1:1 with PBS (without Ca^2+^ and Mg^2+^) and then overlaid on Ficoll-Paque PLUS density gradient media (GE Healthcare Life Sciences, Marlborough, MA, USA). The cells were separated by gradient centrifugation. CD4 T cells were then isolated from the PBMCs using a human CD4 T Cell Isolation Kit (Milteny Biotec, Gaithersburg, MD, USA) by negative selection. The isolated CD4 T cells were cultured in RPMI Medium 1640-GlutaMAX™-I (Gibco™) with 10% heat-inactivated FBS (GE Healthcare), 1 × Antibiotic-Antimycotic Solution (Corning, Corning, New York, NY, USA), and 20 ng/mL IL-2 (Roche, Basel, Switzerland), in a 37 °C, 5% CO_2_ incubator. CD4 T cells from 6 to 8 different donors were cultured with 20 ng/mL of IL-2 (Roche) and activated using 30 μL of ImmunoCult™ Human CD3/CD28 T Cell Activator (STEMCELL Technologies, Vancouver, British Columbia, Canada) per 1 × 10^6^ cells/mL. A total of 2 × 10^6^ CD4 T cells were plated either in the presence or absence of 50 μg exosomes, a dose that was established empirically. After three days of co-culture, CD4 T cells were collected, stained with Live/Dead-Aqua (Invitrogen, Carlsbad, CA, USA), CD4-FITC (BD Bioscience, San Jose, CA, USA), and the activation marker CD25-PE (BD Bioscience), then analyzed by flow cytometry (BD LSRFortessa™ X-20, BD Bioscience) by gating only on live cells. To assess CD4 T cells proliferation, the cells were stained with 1 μM CellTrace™ Carboxylfluorescein Succinimidyl Ester (CFSE) using the Cell Proliferation Kit for flow cytometry (Thermo Fisher Scientific, Waltham, MA, USA). The CFSE-stained CD4 T cells were then activated as described above and co-cultured with 50 μg exosomes. On day 3, the CD4 T cells were harvested, stained with the cell viability marker 7-AAD (Biolegend, San Diego, CA, USA) and CD4-BUV395 (BD Bioscience), and assessed by flow cytometry for CFSE dilution in live CD4 T cells.

### 4.3. Isolation and Purification of Exosomes

Experiments involving exosomes were designed and interpreted in accordance with the Minimal Information for Studies of Extracellular Vesicles 2023 (MISEV2023) guidelines established by the International Society for Extracellular Vesicles (ISEV). These guidelines recommend rigorous characterization of EVs through multiple complementary approaches, including physical, biochemical, and functional validation. In this study, EV preparations were characterized by nanoparticle tracking analysis, transmission electron microscopy, and Western blot analysis using established EV markers (CD9, CD63, CD81, and ALIX), along with negative controls such as calnexin, to confirm the absence of cellular contamination [51].

MSCs isolated commercially from bone marrow (American Type Culture Collection, Manassas, VA, USA) were grown using T-182 Flasks until 80–90% confluence in Mesenchymal Stem Cell Basal Medium supplemented with the growth kit for Adipose, Umbilical, and Bone Marrow-derived MSCs (American Type Culture Collection). Serum full media was subsequently replaced with StemXVivo Serum-Free Human MSC Expansion Media (R&D Systems, Minneapolis, MN, USA) containing one of the following: CQ (10 mM), TX (5 mM), IFNγ (500 units), IFNγ + CQ, or IFNγ + TX. Conditioned media were collected and replaced after 24 h, and collected again at 48 h from two separate flasks, combined, and spun at 300 G for 10 min. Supernatant was then spun at 3000 G for 40 min and again at 16,500 G for 20 min at 4 °C. Supernatant was then filtered through 0.2 mm PES vacuum filtration units (Sartorius, Göttingen, Germany) and concentrated to 5–7 mL using Vivaspin 10 KDa MWCO centrifugal concentrators (Sigma Aldrich, St. Louis, MO, USA). Concentrated media was injected into the AKTA pure 25 M1 Fast Performance Liquid Chromatography instrument (FPLC, Cytiva), and exosomes were separated and purified using the HiPrep 16/60 Sephacryl S400 HR column (GE Healthcare). FPLC-purified PBS fractions containing exosomes were pooled together and concentrated to 300–400 mL using Vivaspin 10 KDa MWCO centrifugal concentrators (Sigma Aldrich). Purified and concentrated exosomes resuspended in PBS were subsequently stored at −20 °C for up to two months. Total protein content of exosomes resuspended in PBS was assessed using Pierce BCA Protein Assay Kit (ThermoFisher, Waltham, MA, USA).

### 4.4. Assessment of Exosomal Markers

Exosome-Human CD63 Isolation/Detection kit (Invitrogen) and Exosome-Human CD81 Isolation/Detection kit (Invitrogen) were used for assessment of CD63 and CD81 protein expression on isolated exosomes, respectively, according to the manufacturer’s protocol. In brief, CD63 or CD81 dynabeads (20 mL per sample) were transferred to Eppendorf tubes and washed twice with 200 mL of isolation buffer (0.2% BSA) using DynaMag 5 (Invitrogen). Samples were removed from the magnet, and 30–50 mg of exosomes were added to each tube, and the total volume was adjusted to 100 mL using isolation buffer. Samples were subsequently incubated overnight at 4 °C using an orbital shaker. The next day, exosomes bound to dynabeads were washed twice with isolation buffer, and 1 mL of PE CD63 Monoclonal Antibody, clone MEM-259 (Invitrogen) or PE Mouse Anti-Human CD81, clone JS-81 (BD Pharmingen) was added to 100 mL of bead-bound exosomes. Samples were then incubated with antibodies for 1 h, washed twice with isolation buffer, and resuspended in 500 mL of PBS. The data were acquired on a BD LSR Fortessa X-20 flow cytometer (BD Bioscience) and analyzed using FlowJo software v.11 (FlowJo, BD Bioscience) and expressed as the mean fluorescence intensity (MFI).

For immunoblotting of exosomal markers, 100 mL of the total protein normalized exosome suspension was transferred to Eppendorf’s containing 25 mL of 5X RIPA lysis buffer (Alfa Aesar) and protease inhibitor cocktail (Sigma Aldrich). After incubation for 20 min on ice with occasional vortexing, 30 mL of 4X Laemli buffer was added to each sample, and the latter were incubated at 90 °C for 10 min. Subsequently, 25 mL of each sample was resolved on a 4–20% Mini-Protean TGX Gel (Bio-Rad, Hercules, CA, USA) using 1X Tris/Glycine/SDS running Buffer (Bio-Rad) at 120 V. After transfer to a 0.2 mm PVDF membrane (Bio-Rad), the later were incubated with one of the following antibodies: CD63 rabbit polyclonal antibody (clone ERP5702, Abcam, Cambridge, UK), CD81 rabbit monoclonal antibody (clone EPR4244, Abcam), TSG101 rabbit monoclonal antibody (clone EPR7130(B), Abcam) or Calnexin rabbit polyclonal antibody (clone ERP3633, Abcam) overnight on an orbital shaker. After incubation with the secondary antibody, membranes were visualized using Azure^®^ c400 Imaging Systems (Azure Biosystems, Dublin, CA, USA).

### 4.5. Assessment of Autophagy Markers

Autophagy markers were assessed using either immunoblotting for LC3B protein (polyclonal antibody, Novus biologicals, Centennial, CO, USA) using standard methods or by CytoID green Autophagy Detection Kit (Enzo Life Sciences, Farmingdale, New York, NY, USA), which measures autophagic vacuoles in lysosomally inhibited live cells using a dye that selectively labels accumulated autophagic vacuoles. Briefly, MSCs were exposed to either TX or CQ for 24 h and subsequently harvested and stained with the dye for 30 min at 37 °C in PBS. After two washes with PBS, the intensity of the green signal was assessed using flow cytometry. Alternatively, cells were opened using RIPA buffer, proteins separated by gel electrophoresis, and the levels of LC3B were probed with the antibody.

### 4.6. Super Resolution Microscopy and Acquisition of Electron Microscopy Images

To achieve high-resolution imaging below the diffraction limit of single extracellular vesicles, a solution containing approximately 1 × 10^8^–1 × 10^9^ exosomes in 1 μL was treated with a combination of staining antibodies. Antibodies provided in the EV profiler Kit (Oxford Nanoimaging, UK) were employed, which included anti-CD9-ATTO488, anti-CD63-Cy3, and anti-CD81-AlexaFluor647. Following the staining process, samples were processed according to the manufacturer’s guidelines to immobilize the stained exosomes on chips provided with the ONI EV profiler kit. Imaging was performed using direct stochastic optical reconstruction microscopy (dSTORM) on a Nanoimager S Instrument (Oxford Nanoimaging, UK). Laser powers of 40%, 20%, and 20% were used for the 488 nm, 561 nm, and 640 nm lasers, respectively. Three fields of view were recorded for each sample. For localization mapping, 2500 images were recorded per channel. Image quantification and colocalization analysis were carried out using the CODI platform (Oxford Nanoimaging, UK), as previously described [52].

For electron microscopy, the exosomes were fixed in 2% paraformaldehyde overnight at 4 °C, loaded onto a Formvar-coated carbon copper grid, fixed with 2% glutaraldehyde, and stained with uranyl acetate. The grids were subsequently viewed at 80 kV in a JEOL JEM-1400 transmission electron microscope (Japan Electron Optics Laboratory, Peabody, MA, USA) and images captured by an AMT BioSprint 12 digital camera (Advanced Microscopy Techniques, Woburn, MA, USA).

### 4.7. Small RNA Library Preparation, Sequencing, and Data Analysis

Exosomes were isolated from serum-free media using the Total Exosome Isolation Reagent kit (Invitrogen) based on the manufacturer’s protocol, with an increase in centrifuge time from 1 h to 1.5 h. Isolated exosomes were subsequently resuspended in 1 mL Takara Lysis Buffer, and small RNAs were isolated using the NucleoSpin miRNA kit (Takara Bio USA, Inc, San Jose, CA, USA). The samples were further purified with the RNeasy MinElute kit (Qiagen, Germantown, MD, USA), with an increase in 100% ethanol volume from 250 uL to 900 uL. Sequencing libraries were prepared using the Illumina TruSeq Small RNA Library Prep Kit (Illumina, San Diego, CA, USA) according to the manufacturer’s protocol. A total of 6 sequencing libraries per run were pooled and normalized to 2 nM and denatured according to Illumina’s NextSeq System Denature and Dilute Libraries Guide. Final pooled libraries were spiked with 1% PhiX (Illumina) as an internal control and loaded at a final concentration of 1.6 pM onto the Illumina NextSeq 500 sequencer. Libraries were sequenced on a 1’50 bp single-end run using the Illumina NextSeq 500 High Output v2.5 Kit (Illumina; 75-cycle, 400 million read flow cell). GEO accession link: https://www.ncbi.nlm.nih.gov/geo/query/acc.cgi?acc=GSE272209 (accessed 23 December 2025). 

After the completion of sequencing, raw sequencing data were transformed to FASTQ format using the bcl2fastq2 v2.20 software (Illumina). An average of approximately 55 million reads was collected for each of the 18 libraries. FASTQ files were processed through an analysis pipeline using the Partek Flow software (Build version 10.0.22.0424; Partek, Chesterfield, MI, USA). Briefly, raw sequencing data were initially checked for quality. Adaptor sequences were trimmed from the sequencing reads. Low-quality reads for either end with Phred quality score below 20 and sequences shorter than 15nt were removed [53]. The trimmed high-quality clean reads were aligned to the human genome (Ensembl GRCh38) using Bowtie-1.0.0, allowing one mismatch and up to 3 genomic locations [54]. MiR expression was quantified by counting the number of reads aligning to the miRBase database, release 22.1 (miRBase v22.1) [55]. Normalization and evaluation of miRNAs differential expression was performed using the R package DESeq2 [56]. For further analysis, we selected miRs that showed statistical significance with an unadjusted *p*-value = 0.05 and the absolute value of Fold Change = 1.5.

### 4.8. RNA Sequencing Validation and Determination of Putative miR Targets

Quality control and quantification of exosomal RNAs were performed by Nova Southeastern University’s Genomic Core Facility via Nanodrop, Qubit RNA High Sensitivity kit (Invitrogen), and Bioanalyzer Agilent Small RNA kit (Agilent Technologies, Santa Clara, CA, USA). cDNA was generated using total RNA isolated from exosomes using the miRNA 1st Strand cDNA Synthesis Kit (Agilent Technologies) per manufacturer’s instructions. The cDNA mixture was quantified on Nanodrop, and all samples were normalized to 500 ng input. qRT-PCR was performed on the Agilent Aria MX using miRNA qPCR Master Mix (Agilent Technologies) per manufacturer’s instructions. miRNA forward primers were designed per manufacturer’s instructions of miRNA qPCR Master Mix (Agilent Technologies): “the forward primer should be identical in sequence and length to the miRNA itself”. The miRNA sequences were obtained from miRBase (https://www.mirbase.org/). Forward primers were synthesized by Synbio Technologies and their sequences used in the assays are as follows: hsa-miR-191: 5′-TCGCTCAACGGAATCCCAAAAG-3′; hsa-miR-99b-5p: 5′-TCGCTCACCCGTAGAACCGAC-3′; hsa-miR-24-3p: 5′-TCGCTTGGCTCAGTTCAGCAG-3′; hsa-miR-625-3p: 5′-TCGCTGACTATAGAACTTTCC-3′; hsa-miR-146b-5p: 5′-TCGCTGCCCTGTGGACTCAGT-3′; hsa-let-7e-5p: 5′-TCGCTTGAGGTAGGAGGTTGT-3′; hsa-miR-100-5p: 5′-AACCCGTAGATCCGAACTTGTG-3′; hsa-miR-10396b-3p: 5′-GGCCCCGGGCCCTCGACCGGAC-3′; hsa-miR-193b-3p: 5′-AACTGGCCCTCAAAGTCCCGCT-3′; hsa-miR-23b-3p: 5′-ATCACATTGCCAGGGATTACCAC-3′; hsa-miR-4516: 5′-GGGAGAAGGGTCGGGGC-3′; hsa-miR-1307-5p: 5′-TCGACCGGACCTCGACCGGCT-3′; RNU6: 5′-CGCAAGGATGACACGCAAATTC-3′; hsa-miR-16-5p: 5′-TAGCAGCACGTAAATATTGGCG-3′. miRNA Universal Reverse Primer (Agilent Technologies) was used as the universal reverse primer, and Human RNU6 and hsa-miR16 were used as housekeeping miRNAs. Each fold change was calculated using the double delta Ct method and normalized separately to both housekeeping miRNAs; the final fold change was calculated as an average of both normalized values.

Putative targets for differentially regulated miRs were determined using the miRNET 2.01 database, which is based on the latest releases from miRNA annotation databases, including miRBase, miRTarBase, TarBase, and HMDD [57,58]. This database also uses human exosomal miRNA annotations from ExoCarta, while the interactions among miRNAs, transcription factors, and genes are obtained from TransmiR 2.0, ENCODE, JASPAR, and ChEA [57]. We analyzed putative miR targets using sets of differentially regulated miRs showing a positive or negative fold increase when compared to controls, using the following parameters: Homo sapiens (species), miRbase ID (identifiers), exosomes (tissues), and miRTarBase V 8.0 (gene targets). Submission of the miRNA data resulted in the output of known gene targets and corresponding biological pathways. Using the ‘Node Explorer’ function, the top 10 gene targets were determined based on the ‘degree’ factor (i.e., the total number of miRNAs from the dataset known to target particular gene) followed by the ‘betweenness’ factor (which measures the number of shortest paths through a node), with the ‘Betweenness’ factor being used a ‘tie-breaker’. Upon creation of the top 10 gene targets for each experimental group, the targeted genes were then categorized into broad groups depending on their various cellular functions or pathway interactions. These pathways and cellular functions were determined using the database GeneCards (www.genecards.org), which allowed classification of gene targets based on their function.

### 4.9. Statistical Considerations

Statistical significance between experimental groups was analyzed using GraphPad Prism’s 10.2.2 ordinary one-way ANOVA with post hoc tests to allow for multiple comparisons. Any data where the *p*-value was found to be higher than 0.05 was labeled as n.s.

## 5. Conclusions

In summary, our study is an attempt to fill a knowledge gap by linking autophagy-modulated exosomal miRNA signatures with functional immune outcomes. The results reveal both the potential and the limitations of using miRNA profiles as predictive biomarkers of exosome efficacy and point to the need for more integrative strategies that consider the full spectrum of exosomal cargo. These insights not only inform the development of more precise exosome-based therapies but also contribute to broader efforts aimed at standardizing research on exosomes and its translation.

## Data Availability

GEO accession viewer for reviewers (confidential): https://www.ncbi.nlm.nih.gov/geo/query/acc.cgi?acc=GSE272209, The following secure token has been created to allow review of record GSE272209 while it remains in private status: cfgrsamadxojxur.

## Supplementary Materials

The following supporting information can be downloaded at: https://www.mdpi.com/article/10.3390/ncrna12010001/s1. Figure S1: The total number of EVs per exposure was quantified, and co-localizing frequencies were determined for CD63, CD81, CD9, or their combinations. Respective MSC treatments are shown at the bottom; Figure S2: Whole immunoblots for exosomal markers; Figure S3: qPCR validation of exosomal small RNA sequencing data was performed to assess randomly selected miR expression. MSCs were treated as indicated on the x-axis; blue bars are values obtained from sequencing, while red bars represent the data obtained from qPCR validation experiments. Each experiment was performed using three independent biological replicates (n = 3); Figure S4: Putative gene targets of upregulated miRNAs targeting genes belonging to the top 5 validated KEGG pathways (*p* < 0.0025). MiRs are labeled as green squares, while the target genes belonging to either cancer-related pathways or immune-related pathways are labeled as blue or red nodes, respectively. Genes in cancer-related pathways are represented as blue nodes, and genes involved in immune/inflammatory pathways are represented as red nodes. Green squares represent miRNAs experimentally found from MSCs treated with CQ (A), TX (B), INFγ (C), CQ + INFγ (D), and TX + INFγ (E).

## Abbreviations

The following abbreviations are used in this manuscript:

MSCs	bone-marrow mesenchymal stromal cells
MIAMI	marrow isolated adult multilineage
IFNγ:	Interferon gamma
CQ	chloroquine
TX	tamoxifen
DMEM	Dulbecco’s modified Eagle’s medium
PBMC	peripheral blood mononuclear cells
LC3	microtubule-associated protein light chain
AGO2	Protein argonaute-2
FDR	false discovery rate
MVBs	multivesicular bodies
mRNA	messenger RNA
miR	micro-RNA
7-AAD	7-aminoactinomycin D
CFSE	carboxylfluorescein succinimidyl ester
PD-L1	programmed death ligand 1
IL	interlukin
IDO	Indoleamine 2,3-dioxygenase
(PG)E-2	prostaglandin E2
TGFβ	transforming growth factor beta
PTGS-2	prostaglandin-endoperoxide synthase-2
iNOS	inducible nitric oxide synthase
