# Supplementary material for: Functional and Molecular Characterization of Extracellular Vesicles Enriched in Exosomes Released by Bone Marrow Mesenchymal Stromal Cells Exposed to IFNγ in Combination with Autophagy Modulators Tamoxifen or Chloroquine
Source: Noncoding RNA. 2025 Dec 24;12(1):1. doi: 10.3390/ncrna12010001 (PMC12821535; doi:10.3390/ncrna12010001)
Supplement: Supplementary file 1 [file ncrna-12-00001-s001.zip › Figure S3.pptx]

## Slide 1
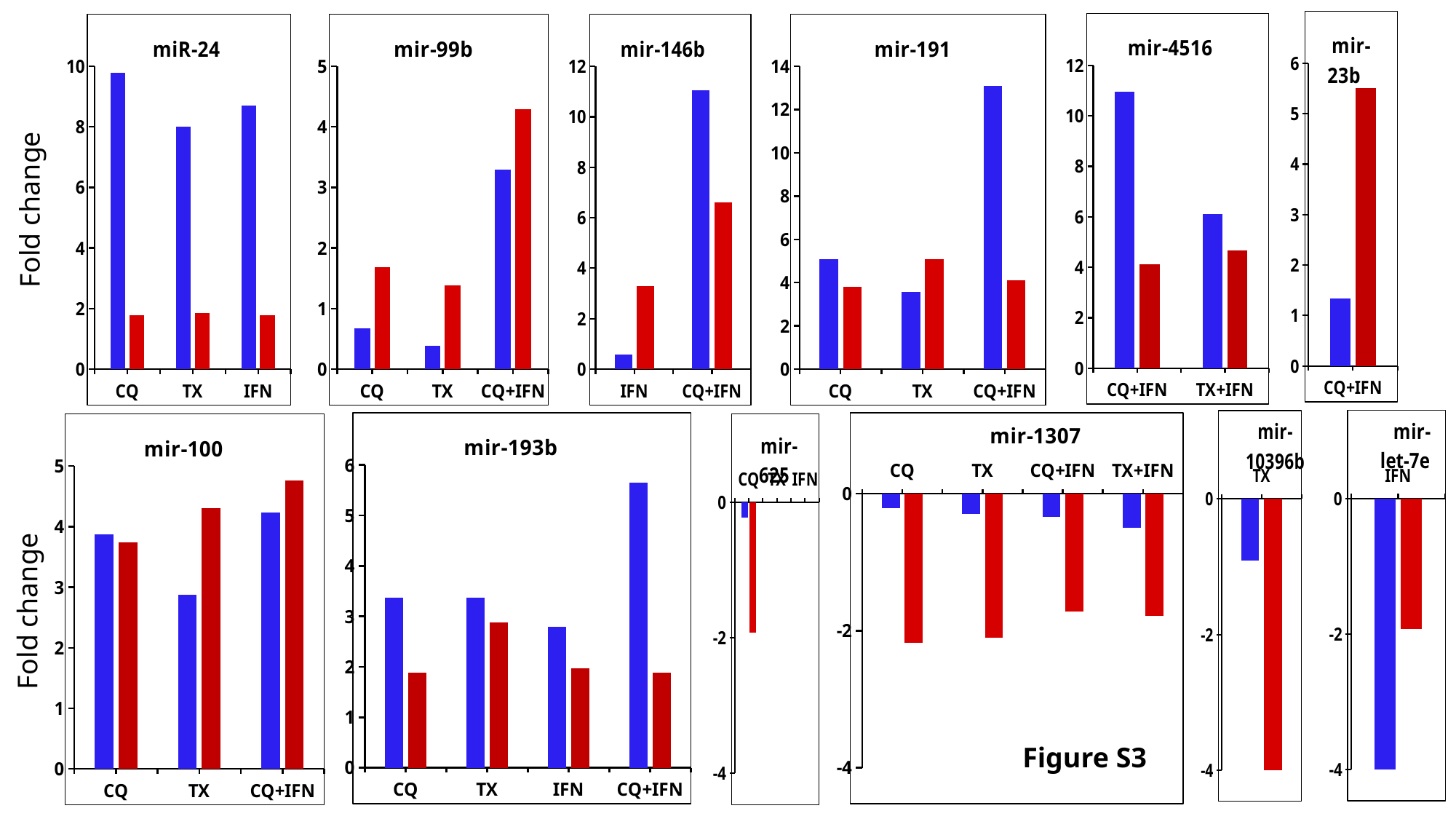

### Chart: mir-23b
| Category | | 5.51 |
|---|---|---|
| CQ+IFN | 1.34 | 5.51 |
### Chart: mir-4516
| Category | | |
|---|---|---|
| CQ+IFN | 10.96 | 4.11 |
| TX+IFN | 6.1 | 4.67 |
### Chart: miR-24
| Category | | |
|---|---|---|
| CQ | 9.787230782924707 | 1.78 |
| TX | 8.014462573119749 | 1.85 |
| IFN | 8.716477644295265 | 1.79 |
### Chart: mir-99b
| Category | | |
|---|---|---|
| CQ | 0.6787070640963109 | 1.6787070640963109 |
| TX | 0.3800299624326495 | 1.3800299624326495 |
| CQ+IFN | 3.2957979645439837 | 4.295797964543984 |
### Chart: mir-146b
| Category | | |
|---|---|---|
| IFN | 0.5910172451393343 | 3.29 |
| CQ+IFN | 11.041983153397792 | 6.62 |
### Chart: mir-191
| Category | | |
|---|---|---|
| CQ | 5.097656543476817 | 3.81 |
| TX | 3.583975441853803 | 5.08 |
| CQ+IFN | 13.078576806523587 | 4.11 |Fold change
### Chart: mir-
let-7e
| Category | | |
|---|---|---|
| IFN | -4.356883869937324 | -1.93 |
### Chart: mir-10396b
| Category | | |
|---|---|---|
| TX | -0.9142153882866892 | -22.8183540044075 |
### Chart: mir-193b
| Category | | |
|---|---|---|
| CQ | 3.37 | 1.88 |
| TX | 3.37 | 2.88 |
| IFN | 2.79 | 1.97 |
| CQ+IFN | 5.65 | 1.88 |
### Chart: mir-1307
| Category | | |
|---|---|---|
| CQ | -0.20890093201513837 | -2.18 |
| TX | -0.29301487455935593 | -2.1 |
| CQ+IFN | -0.3341362353790249 | -1.72 |
| TX+IFN | -0.49938143661644174 | -1.78 |
### Chart: mir-100
| Category | | |
|---|---|---|
| CQ | 3.87 | 3.74 |
| TX | 2.87 | 4.31 |
| CQ+IFN | 4.23 | 4.76 |
[unsupported chart]
Fold change
 Figure S3
