# Supplementary figures and images for: Functional and Molecular Characterization of Extracellular Vesicles Enriched in Exosomes Released by Bone Marrow Mesenchymal Stromal Cells Exposed to IFNγ in Combination with Autophagy Modulators Tamoxifen or Chloroquine
Source: Noncoding RNA. 2025 Dec 24;12(1):1. doi: 10.3390/ncrna12010001 (PMC12821535; doi:10.3390/ncrna12010001)

## Slide 1
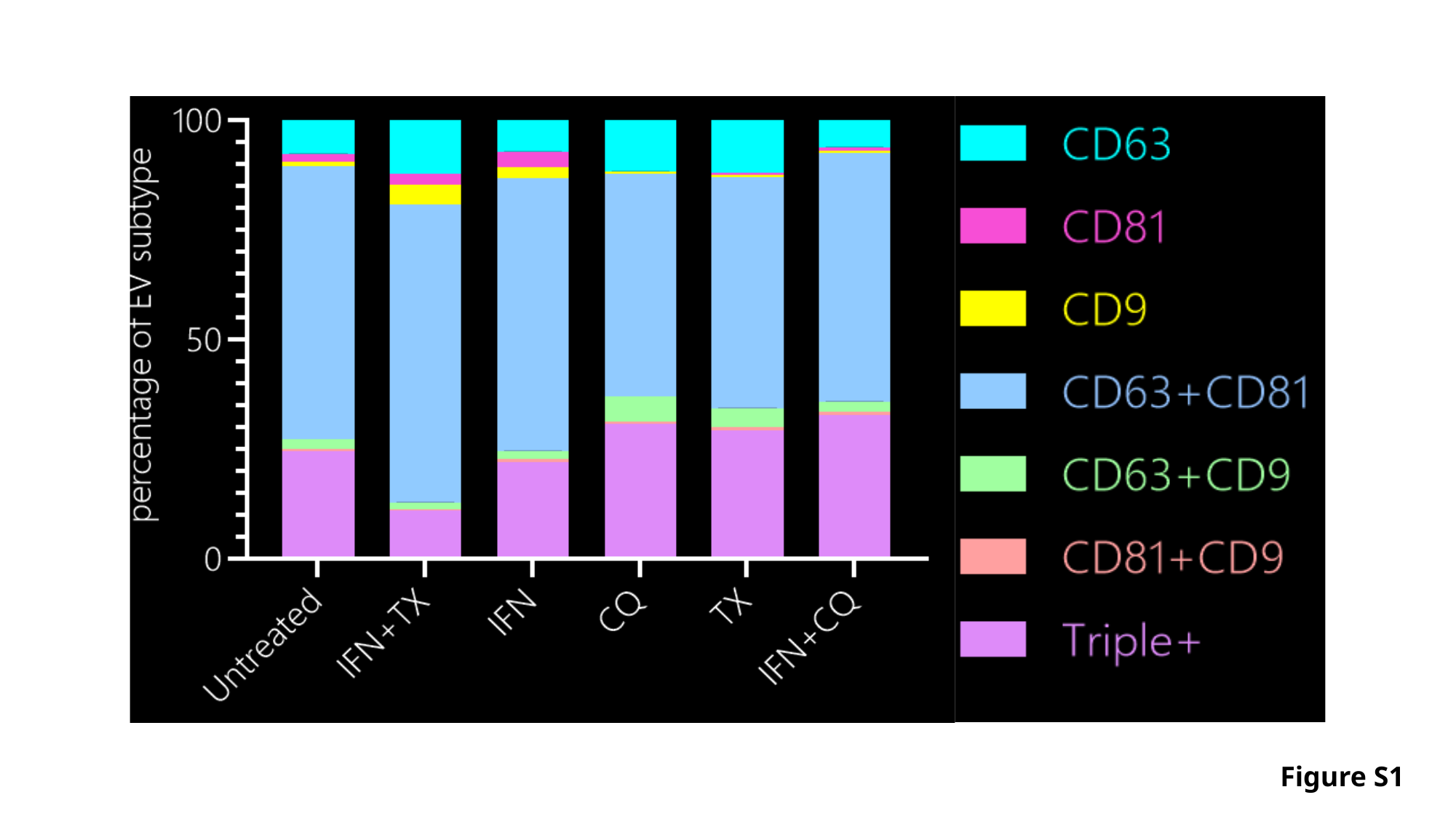

Figure S1

Supplement: Supplementary file 1 [file ncrna-12-00001-s001.zip › Figure S1.pptx]

## Slide 1
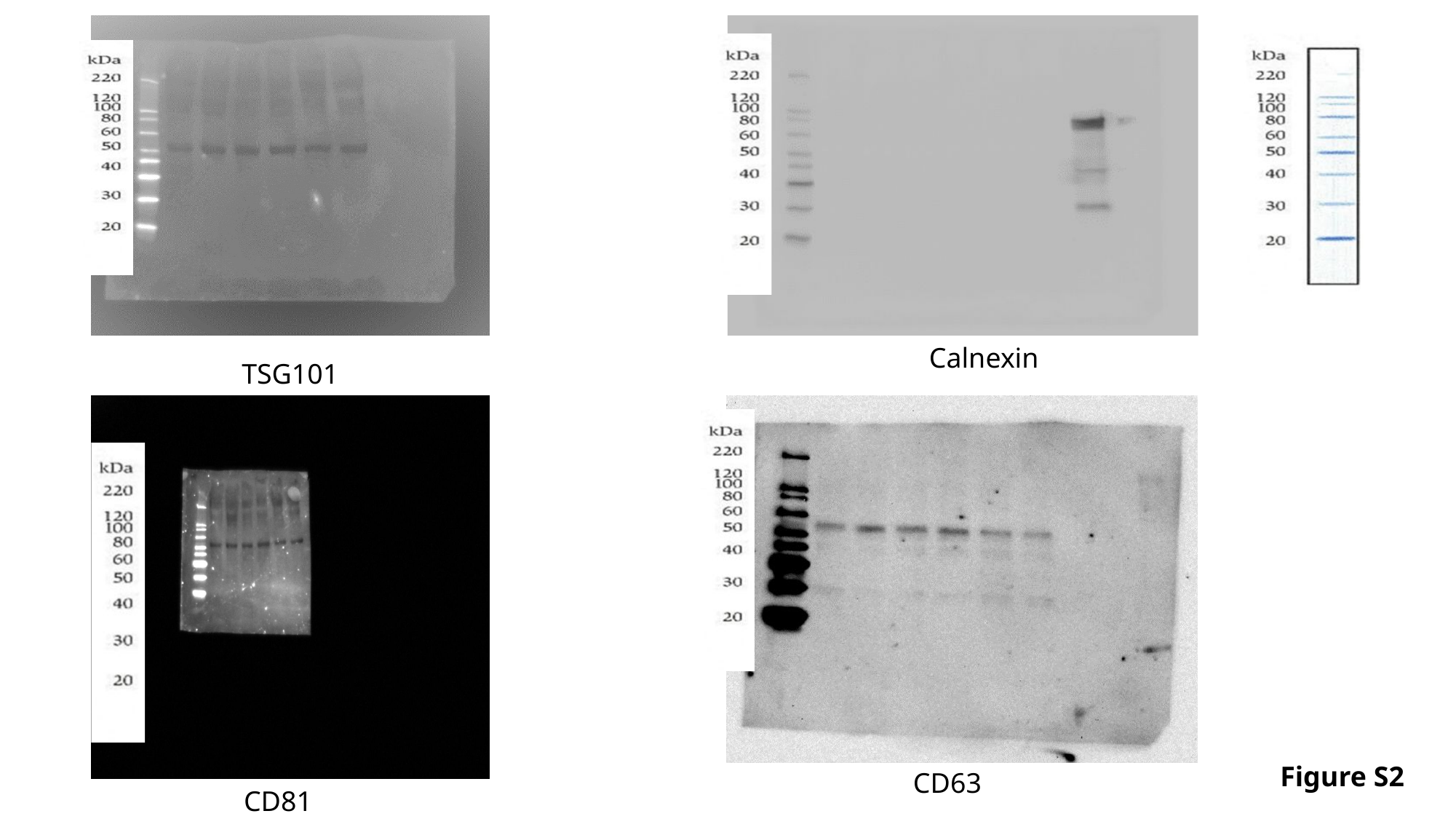

Calnexin
TSG101
 Figure S2
CD63
CD81

Supplement: Supplementary file 1 [file ncrna-12-00001-s001.zip › Figure S2.pptx]

## Slide 1
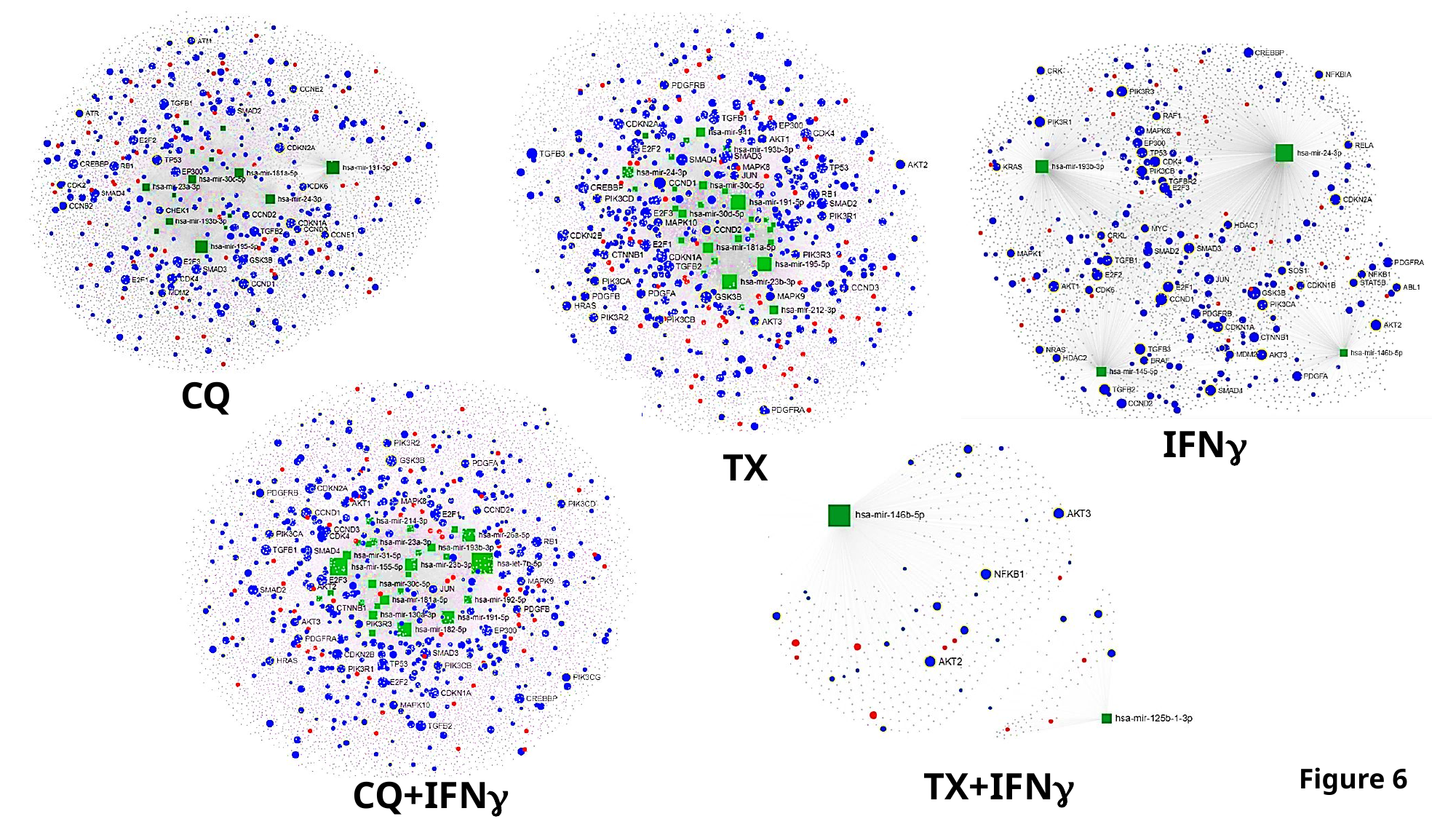

CQ
IFNg
TX
Figure 6
TX+IFNg
CQ+IFNg

Supplement: Supplementary file 1 [file ncrna-12-00001-s001.zip › Figure S4.pptx]
